# Investigating HRV’s contribution to identify exercise pattern and intensity in healthy subjects

**DOI:** 10.1371/journal.pone.0350856

**Published:** 2026-06-11

**Authors:** Clara L. Sanchez, François B. Favier, Arthur Fabre, Alain Varray

**Affiliations:** EuroMov Digital Health in Motion, Université de Montpellier, IMT Mines Ales, Montpellier, France; University of Ljubljana, Medical faculty, SLOVENIA

## Abstract

**Background:**

The ability to discern exercise modalities, defined here as intensity and pattern, holds significant importance in various contexts, particularly in conditions where treatment management is heavily influenced by daily physical activity. The objectives of this study were thus to evaluate the predictive capabilities of heart rate variability (HRV) components for identifying exercise modalities, and to examine whether HRV during exercise exhibits distinctive characteristics depending on the intensity and continuous or intermittent nature of the activity.

**Methods:**

We conducted HRV analysis across different exercises varying in intensity and pattern [two constant-load sessions at 40% (C40) and 60% (C60) of maximal aerobic power, and one intermittent session (INT)], using cycle ergometers with 26 volunteer subjects. HRV was analyzed using time-domain, frequency-domain, and nonlinear measures from data recorded with a Polar H10 Heart Rate Sensor chest strap and processed with Kubios HRV software. Gas exchanges and lactate levels were also measured. HRV data from the final 4 minutes of each exercise session were analyzed using penalized multinomial logistic regression to predict specific exercise modalities performed. Additionally, repeated measures ANOVA were conducted followed by post-hoc tests when appropriate, to assess differences across conditions.

**Results:**

Multinomial logistic regression model demonstrated robust predictive performance, achieving sensitivities and specificities ranging from 88 to 98%. Time-domain (meanRR, SDNN) and nonlinear (DFA-ɑ1) emerged as the retained predictors. ANOVA tests revealed distinct impacts of exercise modality on HRV components: time-domain measures, LF and HF spectral powers, and the nonlinear index DFA-ɑ1 were significantly reduced in C60 compared with C40, while time-domain variability indices, LF and HF spectral powers, and DFA-ɑ1 remained higher during INT exercise than in both other conditions.

**Conclusion:**

HRV emerges as a promising tool for distinguishing between exercise modalities with various intensity and/or continuous or intermittent pattern.

## Introduction

Detecting exercise modalities (pattern and intensity) with simple wearable devices could help in adapting medical treatment for numerous chronic diseases, leading to enhanced patient safety and clinical outcomes [1–4]. The question arises as to whether solutions could be obtained through non-invasive, low-cost, and easy-to-use wearable devices. Heart rate variability [HRV (*i.e.,* time variations between successive heartbeats)] analysis has demonstrated its utility across different fields. Indeed, HRV is recognized as an effective non-invasive biomarker for assessing cardiac autonomic modulation in medical settings, including diagnosis, prediction, disease progression monitoring, and mortality risk in various human pathologies [5–7]. More specifically, in sports science, HRV has also proven effective in analyzing key aspects, particularly in the evaluation of autonomic regulation during exercise [8–15], recovery [8,10,16] and to identify physiological thresholds [17–22]. Research has provided valuable insights, leading to clinical applications, especially in diagnosing conditions such as pathologies, overtraining, and optimizing training intensities [23].

Through these studies, it has been demonstrated that HRV components change as intensity increases, highlighting specific changes in autonomic activity. Consistent findings from these studies show that HRV, traditionally studied in both time and frequency domains [7], significantly decreases during exercise [24]. Time-domain components such as mean RR intervals and their variance decline with increased exercise intensity [13,25]. In the frequency domain, the absolute power of low frequency (LF) and high frequency (HF) spectral components, and thus the total power of HRV, diminish as exercise intensity rises [8,9,11,12,25]. Despite these common trends, the literature remains contradictory due to methodological differences, complicating synthesis [8–10,24]. In addition, the pronounced decrease of these conventional parameters with increasing exercise intensity, prevents a clear differentiation between high and severe intensities, as some variables reach minimal values, close to zero, or form a plateau, thereby limiting in-depth analysis beyond moderate intensity [11,24–26].

As a result, growing interest has emerged in recent years in the literature regarding the use of nonlinear dynamics and chaos theory in the analysis of HRV [23,27,28]. Among these, the nonlinear index based on fractal correlation properties determined by alpha-1 of detrended fluctuation analysis (DFA-ɑ1), has proven promising for assessing the distribution of exercise intensity, including at very high intensities, offering new perspectives for obtaining more detailed insights into complex cardiovascular regulation [29]. During incremental exercise, DFA-ɑ1 systematically evolves in a biphasic manner: from the resting value (typically between 1 and 1.5), stable values, or even slightly increasing up to 1.5, have been reported at very low to light intensities [14,15,30]. Conversely, from moderate to high intensity exercise, DFA-ɑ1 decreases almost linearly to 0.5 or higher [14,15,31,32].

Moreover, HRV during exercise has been predominantly investigated in laboratory settings using standardized continuous or incremental protocols [8–14,30,31,33–36]. These models have primarily enabled the investigation of exercise intensity, which remains the most extensively documented parameter in the literature [24]. In contrast, the influence of other exercise modalities, such as intermittent efforts, has received considerably less attention. As a consequence, knowledge of HRV during intermittent exercise or daily life activities remains sparse [37]. More recently, studies have examined HRV during recovery to infer the training load of continuous and intermittent exercises [38–40]. This indicates that HRV could be used to provide information on the modalities of physical activity sessions, suggesting promising potential for HRV monitoring in daily use.

Thus, the objectives of this study were twofold: i) to explore whether HRV components could serve as predictors of exercise modalities; and ii) to pinpoint components in the time and frequency domains, as well as nonlinear measures that are specifically influenced by exercise modalities, focusing on the exercise pattern (continuous vs. intermittent) and its intensity.

## Methods

### Study participants

**Fig 1:**
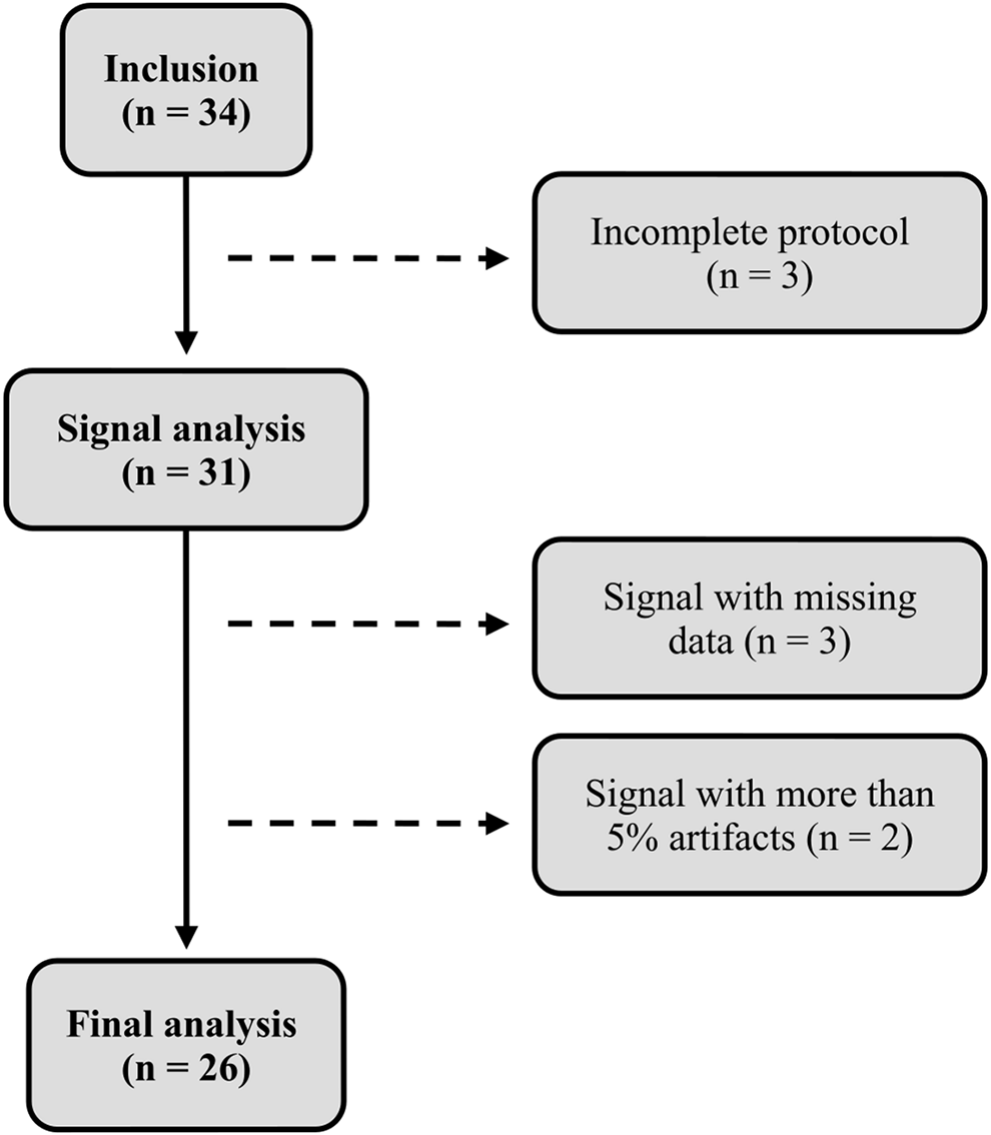
Flow chart for inclusion of subjects in the study.

Thirty-four healthy subjects initially participated in this study, recruited between March and October 2022. After accounting for attrition (Fig 1), the final dataset analyzed included twenty-six subjects (11 males, 15 females). Table 1 provides a summary of the baseline characteristics of these participants. Volunteers were recruited from Montpellier’s Sports Sciences Faculty, the EuroMov Digital Health in Motion laboratory, and professionals in computer science, ensuring a diverse representation of physical fitness levels. Inclusion criteria encompassed ages between 20 and 40 years, absence of chronic diseases, and possession of a valid certificate indicating no contraindication to physical activity. Exclusion criteria comprised inability to perform standard physical exercises or failure to complete all prescribed exercises.

**Table 1:** Participant characteristics.

	Age (year)	Body mass (kg)	Height (m)	BMI (kg/m²)	Indirect $\dot{V}$O_2max_ (ml/min.kg)
n = 26	25.5 ± 4.6	69 ± 11.5	1.71 ± 8.4	23.5 ± 3	42.3 ± 11.3
Range (min – max)	20 - 34	49.9 - 96.9	1.58 - 1.84	19.3 - 32.8	21.3 - 62.3

Data are presented as mean ± standard deviation; BMI, body mass index; $\dot{V}$O_2max_ maximum oxygen uptake.

### Experimental procedures

All participants provided written informed consent prior to their inclusion in the study. The entire protocol was approved by the local Institutional Review Board (IRB-EM 2111A) and was conducted in accordance with the Declaration of Helsinki. The experimental procedure included one indirect fitness evaluation and three physical exercise sessions on a cycle ergometer conducted over four days. All sessions were conducted at the same time of day as the first session, with a minimum of one day between each session. All experiments were conducted at the EuroMov Digital Health in Motion laboratory. Participants were instructed to refrain from strenuous exercise and avoid consuming caffeinated or alcoholic beverages 12–24 hours before each exercise session.

#### Exercise protocols.

Three distinct exercise modalities were evaluated, each lasting 6 minutes. For continuous exercises, an electromagnetically-braked cycle ergometer (Ergoselect 100 P, Ergoline, Germany) maintained constant power output. For intermittent exercise, requiring higher pedaling velocity, a mechanically-braked cycle ergometer (Monark 894E Peak Bike, Sweden) imposed loads irrespective of pedaling speed. Seat height was adjusted to ensure participants’ knees were slightly bent at the lowest pedal position and were comfortable throughout the sessions. During continuous exercises, participants were instructed to maintain a pedal cadence between 50 and 60 rpm. The three modalities were as follows:

- **C40**: Continuous exercise at a power corresponding to 40% of the participant’s indirect maximal aerobic power (MAP_indirect_ see procedure below).
- **C60**: Continuous exercise at a power corresponding to 60% of the participant’s MAP_indirect_.
- **INT**: Intermittent exercise at a pedaling cadence approximately 80% of maximum pedaling velocity (see procedure below) ± 5 rpm, against a braking load equivalent to 6% of the subject’s body mass. It consisted of alternating 10-second exercise and 30-second passive recovery periods, repeated for 6 minutes (9 repetitions total).

#### Indirect determination of $\dot{V}$O_2max_ and maximal aerobic power.

The indirect determination of $\dot{V}$O_2max_ for participants was conducted using the Astrand-Ryhming nomogram [41] with the method validated by Cink and Thomas [42]. This assessment took place on an electromagnetically-braked cycle ergometer (Ergoselect 100 P, Ergoline, Germany). The calculated $\dot{V}$O_2max_ allowed for the individualization of workloads for continuous exercise sessions using the participant’s indirect maximal aerobic power (MAP_indirect_). This was computed using the formula:


$$\begin{array}{c} {\text{MAP}}_{\text{indirect}}=\frac{\left(\text{indirec}t{\dot{V}O}_{\text{2}}ma\text{x}-\text{250}\right)}{\text{10}.\text{3}} \end{array}$$

with MAP_indirect_ in Watts, indirect $\dot{V}$O_2max_ in ml/min, 250 ml/min corresponds to resting O_2_ consumption, and 10.3^−1^ is the Watt equivalent.

#### Determination of maximum pedaling velocity.

The maximum pedaling velocity [V_max_, in revolutions per minute (rpm)] was determined using a Monark 894E Peak Bike cycle ergometer (Sweden). Participants underwent a personalized warm-up against a 2 kg load for 5 minutes. Following a 3-minute recovery period, V_max_ was assessed using a specific protocol: participants performed two 6-second sprints against a braking load set at 6% of their body mass, with a 45-second rest between sprints. Verbal encouragement was provided to maximize pedaling speed during each sprint. V_max_ values were recorded using Monark Anaerobic Test Software (version 3.3.0.0), and the average of the two trials was used.

#### Measurement of RR intervals.

RR intervals were measured using a Polar V800 watch and a Polar H10 heart rate (HR) Sensor chest strap (Polar Electro, Kempele, Finland), both validated for such measurements [43,44]. Given a precision of 2 ms, we can assume that the Polar H10 device operates at a minimum sampling frequency of 500 Hz. The watch was worn on the dominant wrist, and the chest strap was positioned below the sternum after moistening the electrodes for optimal signal conduction. Prior to each session, the watch was synchronized with the Polar Flow mobile application (version 6.4.0), and communication with the chest strap was conducted via Bluetooth. RR interval data were obtained from the Polar Flow website (https://flow.polar.com/diary). Upon retrieval of the RR data, exercise start times were synchronized with OMNIA software (Version 2.1, Cosmed) using Scilab software (version 6.1.1). Data from the final 4 minutes of each exercise session were extracted for subsequent analysis.

#### Processing of RR interval data.

The processed RR interval files were imported into Kubios HRV software (Standard Version 3.5.0, Kubios Oy, Kuopio, Finland) for analysis. The data were interpolated to achieve equidistant sampling at 10 Hz, a frequency chosen to satisfy the Nyquist-Shannon sampling criterion [45]. The trend was removed using the “smoothness priors” method with a smoothing parameter of 6500, adapted to the resampling frequency to achieve a cutoff frequency of 0.039 Hz, ensuring that the spectral components of interest were not altered [46]. Each sequence was filtered using either a “strong” (mean ± 0.15 s) or “very-strong” (mean ± 0.05 s) bandpass filter to eliminate potential artifacts. The level of correction was determined by visual inspection to ensure that only artifacts were removed without altering normal RR intervals [26]. Detected artifacts were automatically replaced using cubic spline interpolation. Sequences containing more than 5% artifacts [47,48] and/or more than 3 consecutive artifacts were excluded from further analysis [26,49], resulting in the exclusion of two subjects (see Fig 1).

RR interval data within the analysis window were processed in both time and frequency domains, following established guidelines [7]. Time-domain analysis included standard deviation of all NN intervals (SDNN) and root mean square of the successive differences of intervals (RMSSD). In the frequency domain, two spectral components were computed: low frequency (LF, 0.04–0.15 Hz), and high frequency (HF, 0.15–0.4 Hz). Additionally, normalized LF and HF powers, the LF/HF ratio, and the peak value of each component (*i.e.,* frequency at which spectral power peaks in Hz), were determined. The HRV spectrum was generated using Fast Fourier Transform (FFT) based Welch’s periodogram method with a 170-second window and 50% overlap. To minimize spectral leakage, Kubios HRV software applied Hanning windows, enhancing spectral resolution [50].

The fractal correlation properties of the RR interval time series were also calculated using the short-term nonlinear scaling exponent of DFA (DFA-ɑ1), with the window widths defined previously (4 < n < 16 beats) [51].

#### Gas exchange analysis.

Oxygen consumption ($\dot{V}$O_2_) and carbon dioxide production ($\dot{V}$CO_2_) were measured breath-by-breath using a K5 metabolic analyzer (Cosmed, Italy) and analyzed with OMNIA software (Cosmed). Prior to each session, the analyzers were calibrated using ambient air (20.93% O_2_ and 0.03% CO_2_) and a calibration gas mixture (16% O_2_ and 5% CO2). Turbine calibration was performed with a 3 L calibration syringe at various flow rates to ensure accuracy. $\dot{V}$O_2_ values were averaged over the final 4 minutes of each exercise modality.

#### Blood lactate measurement.

Fingertip capillary samples were obtained to measure blood lactate levels using the LACTATE PRO 2 analyzer (Axonlab, Switzerland), which has been validated against reference measurements [52]. Before each exercise session, baseline lactate measurements were taken until stable resting values were achieved. Throughout exercise, lactate levels were monitored every minute, extending into the post-exercise period to capture peak lactate concentrations.

#### Experimental protocol.

**Fig 2:**
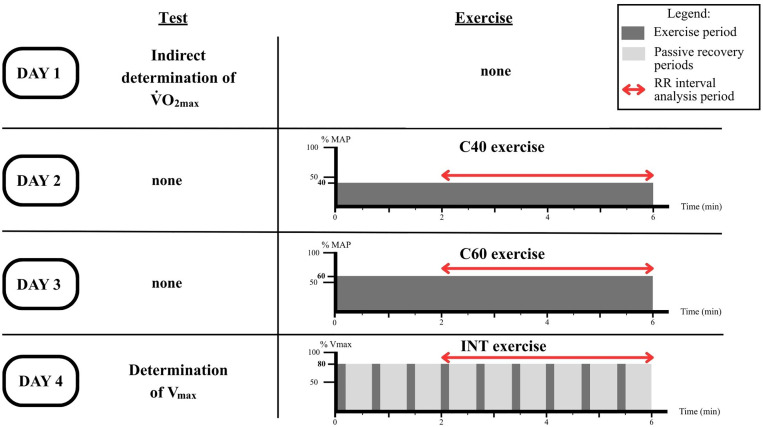
Timeline of the experimental sessions. $\dot{V}$O_2max_: maximum oxygen uptake; V_max_: maximum pedaling velocity; MAP: maximal aerobic power.

The exercise sessions were scheduled across four separate days according to the following plan (Fig 2):

- *Day 1*: Indirect determination of $\dot{V}$O_2max_
- *Day 2*: C40 exercise.
- *Day 3*: C60 exercise.
- *Day 4*: Assessment of maximum pedaling velocity, followed by INT exercise once lactate levels returned close to resting values.

### Statistical analysis

The results are presented as mean ± standard deviation. A significance level of 0.05 was used for all tests. Repeated-measures one-way ANOVA were performed using JASP software (version 0.16.0.0), and multinomial logistic regression were conducted in R (version 4.2.1).

A multinomial logistic regression penalized by an L1 norm (Lasso) [53,54], was applied using a two-step procedure to assess the predictive ability of HRV components. Exercise modality was the dependent variable, defined as a three-level categorical outcome (C40, C60, and INT). Lasso applies a penalty controlled by a regularization parameter λ, which shrinks regression coefficients and sets those associated with weakly informative predictors to zero, thereby enabling automatic variable selection.

In the first step, a variable selection procedure was performed to identify the most stable HRV components. As resampling methods improve the stability of variable selection [55,56], a bootstrap procedure with 200 resamples was applied: for each resample, a multinomial Lasso model was fitted, and variables with non-zero coefficients were retained. Predictors most frequently selected, considered the most robust, were selected for the next step. In the second step, a final multinomial Lasso model was fitted using only the variables selected in the first step. In both steps, the optimal λ was determined through 5-fold cross validation, prioritizing parsimony during variable selection and predictive performance in the final model. Model performance was assessed in terms of sensitivity and specificity using confusion matrices.

The predictive performance and potential overfitting of the final model were evaluated using 5-fold cross-validation performed on a different split of the same data, applying the λ value determined during model fitting. The mean and variability of sensitivity, specificity, accuracy, and area under the curve (AUC) across resampling iterations were reported.

The influence of exercise modalities on HRV measures was assessed using a one-way repeated-measures analysis of variance (ANOVA), with exercise modality (C40, C60, and INT) as the within-subject factor. Before performing the ANOVA, the normality of the within-subject differences between each pair of exercise conditions for each variable was assessed using the Shapiro-Wilk test, and variables that did not satisfy the normality assumption were log-transformed. Sphericity was checked with the Mauchly’s test; when sphericity was violated (p < 0.05), the Huynh-Feldt correction was applied. Post hoc pairwise comparisons were performed using Holm’s test when ANOVA revealed significant effects (p ≤ 0.05).

## Results

### Metabolic profiles of exercise modalities

The exercise modalities demonstrated significant differences in $\dot{V}$O₂ (F = 40.4; p < 0.001; Table 2). Specifically, the C40 condition elicited significantly lower $\dot{V}$O₂ compared to the other modalities, while $\dot{V}$O₂ did not differ significantly between C60 and INT conditions. Maximum lactatemia also varied significantly across exercise modalities (F = 43.5, p < 0.001; Table 2), with peak lactatemia being highest in the INT condition and lowest in the C40 condition. Additionally, there were systematic differences in average power developed during each exercise (F = 204, p < 0.001; Table 2).

**Table 2:** Comparative analysis of exercise modalities: $\dot{V}$O₂, peak lactatemia, and power.

	Exercise modalities			ANOVA		Post-hoc *p value*		
	C40	C60	INT	F	*p value*	C40 *vs.* C60	C40 *vs.* INT	C60 *vs.* INT
**$\dot{V}$O₂(ml/min.kg)**	22.9 ± 6.8^a^	32.3 ± 10.4^b^	31.4 ± 7.8^b^	40.4	<0.001	<0.001	<0.001	0.423
**Peak lactatemia(mmol/L)**	4.0 ± 1.7^a^	8.4 ± 3.2^b^	10.2 ± 3^c^	43.5	<0.001	<0.001	<0.001	0.007
**Power(W)**	104 ± 36^a^	155 ± 54^b^	427 ± 129^c^	204	<0.001	0.007	<0.001	<0.001

Data are presented as mean ± standard deviation; $\dot{V}$O₂, exercise oxygen consumption.

Different superscript letters indicate significant differences between conditions (post hoc test, p < 0.05). Letters shared by groups indicate no significant difference.

### Prediction of exercise modalities using HRV data

During the selection procedure, three variables were retained in more than 95% of the resampling iterations: meanRR, SDNN, and DFA-α1. These variables were therefore included in the final model.

In the fitted model, sensitivity reached 88.5% for C40, 96.2% for C60, and 92.3% for INT, while specificity was 96.2% for C40, 94.2% for C60, and 98.1% for INT (Table 3).

**Table 3:** Confusion matrix and classification performance (sensitivity and specificity) for each modality in the fitted model.

Observed modalities	Predicted modalities			Sensitivity	Specificity
	C40	C60	INT		
**C40**	23	2	1	88.5%	96.2%
**C60**	1	25	0	96.2%	94.2%
**INT**	1	1	24	92.3%	98.1%

Predictive performance assessed through 5-fold cross-validation was stable across folds, with limited variability and values comparable to those obtained in the fitted model (Table 4).

**Table 4:** Cross-validated predictive performance metrics (mean ± standard deviation) of the fitted model.

Metric	Mean ± standard deviation
**Sensibility**	89.8 ± 3.4%
**Specificity**	94.8 ± 1.8%
**AUC**	98.2 ± 2.3%
**Accuracy**	89.7 ± 3.5%

### Analysis of heart rate variability

Time-domain, frequency-domain, and nonlinear measures showed significant differences between exercise modalities (Table 5).

**Table 5:** HRV results and comparisons by exercise modalities in the time- and frequency-domain and nonlinear index.

	Exercise modalities			ANOVA		Post-hoc p value		
	C40	C60	INT	F	p value	C40 *vs.* C60	C40 *vs.* INT	C60 *vs.* INT
***Time domain***								
**MeanRR (ms)**	462 ± 38.0^a^	385 ± 22.3^b^	386 ± 35.0^b^	94.4	<0.001	<0.001	<0.001	0.898
**SDNN (ms)**	5.89 ± 2.28^a^	2.57 ± 0.79^b^	8.96 ± 3.66^c^	43.2	<0.001	<0.001	<0.001	<0.001
**RMSSD (ms)**	3.55 ± 1.05^a^	2.37 ± 0.43^b^	3.48 ± 0.72^a^	15.7	<0.001	<0.001	0.991	<0.001
***Frequency domain***								
**LF (ms²)**	16.6 ± 12.1^a^	1.89 ± 1.85^b^	12.1 ± 14.3^c^	34.8	<0.001	<0.001	0.045	<0.001
**HF (ms²)**	5.95 ± 7.71^a^	0.91 ± 1.71^b^	3.16 ± 5.41^c^	24.3	<0.001	<0.001	0.001	0.002
**LF (nu)**	78.7 ± 15.1^ab^	67.9 ± 24.7^a^	83.2 ± 14.9^b^	3.79	0.04	0.081	0.629	0.038
**HF (nu)**	21.1 ± 15.0^ab^	31.8 ± 24.4^a^	16.7 ± 14.7^b^	6.15	0.004	0.147	0.147	0.003
**LF/HF ratio**	5.73 ± 3.78^ab^	4.64 ± 4.71^a^	9.71 ± 8.71^b^	5.91	0.005	0.1	0.164	0.004
**LF peak (Hz)**	0.07 ± 0.02^a^	0.06 ± 0.02^ab^	0.05 ± 0.004^b^	7.67	0.002	0.094	<0.001	0.094
**HF peak (Hz)**	0.31 ± 0.09^a^	0.3 ± 0.1^a^	0.24 ± 0.1^b^	5.36	0.01	0.546	0.01	0.033
***Nonlinear index***								
**DFA-ɑ1**	1.17 ± 0.23^a^	0.7 ± 0.25^b^	1.16 ± 0.3^a^	41.3	<0.001	<0.001	0.889	<0.001

Data are presented as mean ± standard deviation; SDNN, standard deviation of all NN interval; RMSSD, root mean square of the successive differences of intervals; LF, low frequency; HF, high frequency.

Different superscript letters indicate significant differences between conditions (post hoc test, p < 0.05). Letters shared by groups indicate no significant difference.

In the time domain, mean RR was significantly higher in the C40 condition compared to both C60 and INT (F = 94.4; p < 0.001). SDNN values differed significantly across the three modalities (F = 43.2; p < 0.001), with the highest values observed in INT and the lowest in C60. RMSSD was significantly lower in C60 compared to the other conditions (F = 15.7; p < 0.001).

In the frequency domain, both LF and HF spectral power differed significantly across all three modalities (F = 34.8; p < 0.001 and F = 24.3; p < 0.001, respectively) with the highest values observed in C40 and the lowest in C60. Additionally, normalized powers as well as the LF/HF ratio showed significant differences between the C60 and INT modalities. LF peak was significantly higher in C40 compared to INT (F = 7.67; p = 0.002). HF peak was significantly lower in INT condition compared to both C40 and C60 (F = 5.36; p = 0.01).

For the nonlinear index DFA-ɑ1, values were significantly higher in C40 and INT conditions compared to C60 (F = 41.3; p < 0.001).

## Discussion

This study aimed to evaluate whether HRV components could be used to predict exercise modalities and to identify HRV components influenced by exercise modalities. Our results show significant differences in several HRV components across the different modalities. In addition, our predictive analyses indicate that distinct HRV components allow good discrimination between the considered exercise modalities, characterized by different patterns and intensities.

### Predictive accuracy for identifying exercise pattern and intensities using HRV components

The prediction models derived from multinomial logistic regression demonstrated strong discriminative performance in distinguishing exercise modalities based on HRV, with sensitivities and specificities ranging from 88 to 98%. The selection procedure identified three robust predictive variables: the time-domain variables meanRR and SDNN, and the nonlinear index DFA-ɑ1.

The effects of exercise intensity on HRV have been extensively studied in the literature, with detailed analyses of changes observed in time-domain, frequency-domain, and nonlinear measures. However, despite this wealth of data, no study, to our knowledge, has explored the use of statistical models, such as multinomial logistic regression, to assess the discriminative power of HRV components in predicting exercise modalities.

From an exploratory perspective aimed at detecting exercise modalities, it is essential to combine both linear and nonlinear analyses of HRV. Integrating these complementary approaches within mathematical tools or models allows for more effective exploitation of the information embedded in the HRV signal, by simultaneously capturing both the quantity and the structure of variability. Indeed, during exercise, linear indices (time- and frequency-domain measures, which quantify variability) are particularly more sensitive to low-intensity levels than nonlinear measures, where the most prominent changes are observed [24,57,58]. However, as exercise intensity increases, linear HRV analysis typically shows a marked, almost exponential decrease in global variability. Consequently, these indices become less informative, or even unusable, at high intensity. In turn, nonlinear indices are able to detect changes in HRV dynamics even at high intensity [15]. Thus, the combined use of linear and nonlinear indices appears necessary to effectively discriminate between different exercise modalities. Moreover, the strong discriminative performance of the multinomial logistic regression model suggests that they capture underlying signatures that are relatively robust to individual variability, even though previous studies have reported mixed findings during exercise [23,59,60].

This statistical model could thus provide information on the nature of the exercise performed through a simple calculation, without the need for complex equipment that is not compatible with daily life. Furthermore, the application of HRV in determining activity modalities opens multiple perspectives and opportunities across both sports and medical fields. This knowledge is crucial for optimizing management strategies, whether for enhancing performance or adapting therapies effectively. This approach is particularly relevant in real-life conditions, especially for patients with type 1 diabetes, for whom different types of physical activity can have opposing effects on glycemic dynamics, making insulin therapy adjustment particularly complex [61].

### Effects of exercise intensity (C40 *vs.* C60)

Increased intensity showed the expected reduction in HRV, expressed in both the time and frequency domains. Higher intensity C60 exercise was associated with a lower mean RR and reduced overall variance, as measured by the SDNN, in line with prior research showing a decrease as exercise intensity increases [9,13,14,25]. The reduction in RMSSD with increased intensity also aligns with established literature [15,26], indicating alterations in cardiac parasympathetic modulation [6,24]. As a consequence of reduced RR signal variance with increasing intensity, the absolute power of each spectral component also declined [8,9,13,59], particularly the HF component, recognized as indicative of vagal withdrawal [8,10,35,59]. Our study validates these observations, with markedly lower LF and HF spectral powers in the higher intensity, approaching values close to 0, similar to previous findings [11,14,26]. These low spectral power values during intense exercises suggest lower effective autonomous heart rate (HR) control at higher intensities, and the residual variability likely influenced by non-neuronal mechanisms such as the mechanical impact of hyperventilation on the sinus node [12,25,33,62]. Our results support these mechanisms, since HF expressed in normalized units showed a non-significant increase with intensity, as reported by others [9,11,15,25,33]. These studies have demonstrated that a progressive increase in HFnu can lead to an inversion of the LF/HF ratio at higher intensities. Some authors have suggested that this inversion of the ratio could reflect the attainment of the ventilatory threshold [25]. However, we did not observe such an inversion, which may be attributed to several potential factors, such as exercise intensity and/or physical fitness level. For instance, Casties et al. (2006) found the inversion occurring beyond 70% of $\dot{V}$O₂_max,_ while Giles and Draper (2018) did not observe any reversal even above 80% of $\dot{V}$O₂_max_. Moreover, the relative intensity required to reach the ventilatory threshold, and potentially the inversion of the ratio, varies depending on the fitness level of the subjects, as observed in an earlier study [33]. Methodological differences between studies may also contribute [10], thus explaining the variability in results.

A significant decrease in HR correlation properties, as measured by DFA-ɑ1, was observed in C60 exercise, consistent with previous studies reporting a decline in complexity with increasing intensity [14,15,31]. The DFA-ɑ1 values for C40, close to 1, are indicative of high fractal correlation properties and high complexity, typically associated with low-to-moderate intensity exercise [51,63]. This value reflects a HR signal behavior similar to that of 1/f noise, suggesting an optimal adaptive capacity of the cardiovascular system, based on a dynamic balance between complete predictability and randomness [29]. In contrast, the values around 0.7 during C60 indicate a signal behavior between correlated and anti-correlated properties, with reduced complexity, describing moderate- to high-intensity exercise [14,29,32]. These observed correlation behaviors in DFA-ɑ1 values would be due to changes in autonomic balance, reflecting the complex interaction between its parasympathetic and sympathetic branches [21,24,64]. Based on resting DFA-ɑ1 values generally between 1 and 1.5, an increase in DFA-ɑ1 during low-intensity exercise reflects vagal withdrawal [14,30]. Conversely, the inflection point where DFA-ɑ1 decreases, reflects the dominance of sympathetic activity, which increases following vagal withdrawal [14]. This decline could also be influenced by the respiratory sinus arrhythmia [65]. Finally, this loss of fractal complexity has been described as depending more on global parameters of physiological demand than on a simple absolute increase in HR [32,66].

### Effects of exercise pattern (INT *vs.* C40 – C60)

The C60 and INT exercises resulted in similar mean RR values. However, SDNN and RMSSD were significantly higher during the INT exercise, potentially due to rapid fluctuations in cardiovascular activity during alternating exercise and recovery periods. During exercise, there is a decrease in parasympathetic activity and an increase in sympathetic activity, whereas during recovery, the opposite occurs [24]. Previous research has shown rapid vagal reactivation within seconds to minutes during immediate recovery, indicated by an increase in RMSSD [67,68]. The inclusion of recovery periods in the INT condition likely contributed to the significantly higher RMSSD compared to C60. RMSSD in the INT condition did not significantly exceed that of C40, probably due to the inherently greater variability at lower exercise intensities.

Similarly, despite similar mean RR values, LF and HF spectral powers were significantly higher in the INT condition compared to the stable C60 condition, consistent with previous findings [37]. Their study compared steady-state cycling with intermittent judo activity in 4-minute intervals. HRV appears markedly higher during intermittent exercise compared to the C60 condition, despite comparable mean HR. This was unexpected as a decreased HRV should logically be associated with an increase in HR when intensity increases [35]. In addition, Cottin et al. (2004) noted comparable LF and HF distributions in normalized units between the two activity types, as observed in our study, and concluded that the exercise pattern does not significantly alter autonomous HR control under similar training loads [37].

Despite the high intensity of the INT exercise, a DFA-ɑ1 value greater than 1, and also significantly higher than C60, was obtained. This result contrast with expectations from the literature, where values above 1 are generally associated with low intensity, or even rest [14,32,63]. The INT exercise protocol appears to reflect a HR dynamics behavior characterized by high complexity, suggesting an optimally adaptive cardiovascular system. This divergence could be explained by the nature of the exercise, which involves alternating phases of effort and recovery. To our knowledge, only one study has analyzed DFA-ɑ1 during intermittent exercise, but it was calculated only during the exercise phase [69], the obtained values (around 0.6) were therefore consistent with the exercise intensity and the data reported in the literature. While its discriminatory value in relation to exercise intensity has already been established, our results suggest that DFA-ɑ1 could also be relevant for differentiating exercise patterns, particularly between continuous and intermittent efforts.

It should be noted that our analysis was performed using a one-way ANOVA (exercise modalities: C40, C60, and INT). Post-hoc comparisons revealed some differences that can be attributed to intensity (e.g., C40 vs C60, same pattern), or to exercise pattern (e.g., INT vs C60, similar intensity). However, due to the absence of a low-intensity INT modality, this approach does not allow for formal testing of the independent effects of intensity and pattern, nor their interaction. This limitation should be considered when interpreting the results, and conclusions should remain cautious regarding the attribution of observed differences to each modality.

### Methodological considerations and limitations

It should be clarified that meanRR reflects the average duration of RR intervals and is therefore not a variability metric *per se*. However, meanRR is recognized by the Task Force on HRV (1996) as a descriptive measure of the RR interval time series, since all HRV indices are mathematically derived from this series. Moreover, meanRR, being inversely proportional to HR, provides essential contextual information about the underlying chronotropic state, which conditions the interpretation of variability-based metrics. Although it does not quantify variability structure, its inclusion as a covariate in multidimensional analyses is methodologically appropriate. In our study, meanRR contributed meaningfully to the multinomial logistic regression model when combined with time-domain and nonlinear HRV components.

To optimize predictive performance while limiting the risk of overfitting, we used a multinomial logistic regression penalized with Lasso. This approach is particularly relevant in contexts where several variables may be redundant or only weakly informative, and where reducing model complexity improves robustness, generalizability, and computational efficiency [70]. To further assess the stability of the model and limit the risk of overly optimistic performance estimates, we examined the variability of the results using a k-fold cross-validation. The average performances remained similar, with low variability across resampling iterations, suggesting a stable model with limited overfitting. Ideally, a large dataset would allow a clear separation between training, validation, and test sets, but in a small-sample context, setting aside an independent test set would result in a substantial loss information, which justifies the use of cross-validation strategies [71]. However, these promising discriminative performances do not replace the need to confirm their predictive ability using larger samples, independent datasets and a broader range of physical activities (e.g., running, swimming). Exploring sex-specific effects could provide additional insights and represents a valuable future research direction.

FFT was used to obtain the frequency content in the whole signal for predictive purposes, contrary to its conventional use, namely the physiological interpretation of HRV on stationary signals [72]. Stationarity was therefore not verified in this context. However, analyses were conducted on the last four minutes of the signals to exclude the initial HR drift at the start of exercise. Additionally, we performed additional analyses using the full six-minutes signals and found prediction qualities comparable to those obtained with the last four minutes with sensitivity and specificity ranging from 88 to 98%.

Furthermore, several window widths for the Welch periodogram have been tested, and no impact was observed on the predictive performance of the models. In fact, most of the predictors included (time-domain and nonlinear measures) are not derived from the FFT and are therefore not affected by the window width used.

One strength of the present study is that data were obtained from people with a variety of physical activity profiles. HRV analysis can thus help building models able to detect the exercise practiced whatever the fitness level of the subject. Nevertheless, the choice of performing INT exercise against a load of 6% of body mass was not adapted to every participant, often too high for the less trained. It would have been preferable to determine the force-velocity profile in order to set a common % velocity with a load adapted to each participant.

As for the experimental protocol, the order of the exercises was not randomized due to methodological constraints. However, given the short and sub-maximal nature of the exercises, the minimum 24-h recovery period between sessions, and the deliberate scheduling of the most demanding exercise last, it is highly unlikely that an order effect influenced the observed physiological responses. In addition, it would have been interesting to compare two different intensities for intermittent pattern.

## Conclusion

In conclusion, this study represents the first attempt to determine the exercise modalities in relation to HRV responses. HRV, particularly the integration of temporal and fractal indices, emerge a promising tool for predicting between different physical activity patterns and intensities, particularly when using wearable devices. Future research should explore its applicability across various activities (e.g., running, swimming) and in daily life scenarios. Confirming the external validity of multinomial logistic regression prediction models with additional datasets will be essential for broader applicability.
