# Supplementary material for: Investigating HRV’s contribution to identify exercise pattern and intensity in healthy subjects
Source: PLoS One. 2026 Jun 11;21(6):e0350856. doi: 10.1371/journal.pone.0350856 (PMC13258023; doi:10.1371/journal.pone.0350856)
Supplement: Attachment — Submitted filename: Response to Reviewers.docx [file pone.0350856.s002.docx]

We sincerely thank the reviewers for the time they devoted to reading and evaluating our manuscript. Their comments and suggestions were extremely valuable and have allowed us to significantly improve the paper. Please find below our point-by-point responses (black) to the reviewers’ comments (green). The line numbers indicated in our responses refer to the version of the manuscript with tracked changes.

[Reviewer #1:	1](#_Toc221769073)

[Reviewer #2:	4](#_Toc221769074)

[Reviewer #3:	9](#_Toc221769075)

# Reviewer #1:

*1) Purpose - Generally speaking, "exercise modalities" refer to the mode under which the exercise is done, such as cycling, running, resistant training, etc. While some may include session pattern or intensity within "modality" I believe that the title could be slightly misleading to the general reader. Maybe specifically referring to "identifying exercise pattern and intensity".*

We carefully considered the choice of this term. Initially, we believed that providing its explicit definition at the beginning of the article would eliminate any ambiguity. However, in light of your comment and that of Reviewer 3, which suggested a shorter title, we have decided to modify and shorten it.

*2) Introduction - in the first paragraph, you refer to the influence of exercise on drug pharmacokinetics and then never mention it again. It's not specifically related to the purpose of this paper either. If this is the main motivation, I believe that you should change your purpose statement. If not, this should only be mentioned in the intro or discussion as a practical application of the findings of this manuscript. The introduction reads as though we should be paying close attention to drug pharmacokinetics.*

We thank the reviewer for this relevant remark. Your comment is in line with that of Reviewer 3. This paragraph has been moved to the Discussion section (lines 333– 336).

*3)* *The questions from line 47-51 needs clarification. When would clinicians be monitoring exercise and not be able to distinguish between exercise patterns and intensity? Why is HRV a tool that should/could be used here when other tools cannot?*

We thank the reviewer for this comment. We aimed to emphasize that the automatic identification of exercise modalities primarily addresses a need of the patient, rather than the clinician, in daily life conditions, where accurate prediction of forthcoming physical activity is difficult.

In certain chronic conditions, such as type 1 diabetes, the nature of physical activity has a major and sometimes opposite impact on glycemic kinetic, making the adjustment of insulin therapy particularly complex (Riddell et al.,2017^[1]^, Reddy et al., 2019^[2]^). The ability to identify these exercise modalities under real-life conditions is therefore essential for safe and effective treatment management. In this context, HRV represents a relevant tool, as it can be measured using simple, non-invasive wearable devices suitable for continuous monitoring.

*4) When talking about your exercise protocols and MPV protocol, if they have been used elsewhere or are inspired by protocols used elsewhere you need to cite them.*

The exercises included in this protocol were not directly derived from existing protocols, since our objective was not to validate specific modalities but rather to introduce heterogeneity in the exercises. VO_2_ and lactate measurements confirm that this variability was successfully achieved.

*5) 2.2.8 - If you did all of these protocols in the same order, you run into the possibility of your results occurring due to ordered effects. There needs to be an explanation of either why you couldn't counter-order the exercise protocols to prevent this or why it was better to do it the same order every time. Especially with HRV, there is likely to be an ordered effect due to fatigue unless you've controlled for the history with the subjects training every day.*

The order of the exercises was not randomized for methodological reasons. Session day 1 was necessarily conducted first to determine the workloads for continuous-type exercises. Similarly, the organization of session day 4 was a deliberate decision, as the INT exercise was the most demanding and likely to induce prolonged fatigue. It is why it was scheduled last. Among the remaining exercises, only an interchange between the C40 and C60 exercises would have been feasible. Nevertheless, an order effect is unlikely, as these exercises were short (6 minutes), sub-maximal, and separated by at least 24 hours of recovery. Participants refrained from engaging in any intense physical activity during the 12 to 24 hours preceding each session.

Based on these considerations, it is highly unlikely that the order of exercises influenced the observed physiological responses. This has been clarified in the Discussion section (lines 463 – 466).

For information, please find below a global overview of pre-exercise lactatemia for the different exercise conditions: C40 = 1.47 ± 0.28 mmol/L, C60 = 1.49 ± 0.43 mmol/L, and INT = 1.62 ± 0.38 mmol/L (F=2.03; p=0.142).

*6) 2.3 - A problem with your statistical approach is that you have 2 logistic regression comparisons of exercise pattern, (c40 vs INT & c60 vs INT), but only 1 comparison of intensity (c40 vs c60). This should be consistent. Additionally, I highly recommend conducting a multiple-logistic regression as opposed to three separate logistic regression since each of them are aiming to determine the same purpose and it would make your data stronger.*

We thank the reviewer for the suggestion to use multinomial logistic regression models. We have taken his recommendation into account and updated our statistical analyses accordingly by implementing a penalized multinomial logistic regression approach, which strengthens this part of the work.

*6.1) 2.3 - You ran the normality after conducting the ANOVA, (it seems), but did you determine the normality of the variables beforehand? Few HRV variables are distributed normally, and therefore most studies apply a natural-log transformation to metrics that violate normality and then run the stats on them. This allows for the inferences to be more robust and strong.*

We apologize for the lack of clarity. Normality of pairwise differences between conditions was verified prior to performing the repeated-measures ANOVA, following methodological recommendations for determining the use of parametric or non-parametric tests. This has been clarified in the Methods section (line 237). Nevertheless, to address your comment and that of Reviewer 3, we applied a logarithmic transformation to variables that did not meet the normality assumption, in order to approximate normality and allow the exclusive use of parametric ANOVA. This modification has been added to the Methods section (line 239).

Importantly, analyses performed after the transformation yielded the same results.

*7) On Table 2 and 6 you need to include some form of star or asterisk to denote statistical differences between values.*

We apologize for this oversight. Superscript letters indicating significant differences have been added to the tables, and the legends have been updated accordingly.

*8) Because of the way that high/short intervals of power can drastically effect the average power numbers, and the physiological response is not well related to this, I would recommend reporting average AND normalized power from each exercise test.*

We are sincerely sorry, but we are not sure to well understand the meaning of your comment. If the concern relates to the potential inclusion of rest phases in the calculation of power, we confirm that only the active exercise phases were considered. If the suggestion concerns normalizing power, we would appreciate further guidance on the preferred normalization method. In our view, body weight does not directly influence power in the context of the exercises used in this study. We would be glad to make the necessary adjustments once your request has been clarified.

*9) Due to the non-normal distribution of HRV metrics, and the few post-hoc p-values from the ANOVA, it seems as though most results did not show significant differences between c40 and INT, though differences were more consistent in c60 vs INT. This seems to call into question the HRV metrics' ability to determine the differences between exercise pattern, in contrast with the logistic regression results. This should be clarified in the discussion.*

The absence of a significant difference for a variable between certain conditions in an ANOVA does not imply that the variable cannot be predictive in a logistic regression model. These two types of analyses are complementary but provide different informations: ANOVA assesses each variable individually across all conditions, whereas logistic regression evaluates the combined predictive capacity of variables taken together. Therefore, the apparent discrepancies between ANOVA and logistic regression simply reflect these methodological differences.

*10) It is very important to address the fact that 2 of your 3 individual logistic regressions had MeanRR interval as the first predictor, and MeanRR is not specifically a measure of heart rate VARIABILITY. The practicality of MeanRR is the same as HR. Additionally, there is already a significant amount of research that shows that HR itself can distinguish between exercise intensity and pattern (when normalized). You should include HR as one of your metrics to determine if HRV can assist in distinguishing between these factors of exercise OVER AND ABOVE HR, and then convince us why HRV is important as a separate predictor/determinant of exercise pattern and intensity.*

We thank the reviewer for raising this point.

Although meanRR is not a variability metric in itself, its inclusion is methodologically justified because all HRV measures are mathematically derived from the series of RR intervals, and meanRR (inversely proportional to heart rate) reflects the baseline chronotropic state that can condition the interpretation of HRV metrics. Specifically, the average RR interval provides essential contextual information about physiological status even if it does not itself express the structure of variability. Thus, meanRR used as a covariate in a multidimensional domain analysis is fully appropriate. Our results show a good predictive capacity in logit when this variable is associated with temporal and non‑linear indices of HRV. This result is consistent with the rationale outlined above. This point has been clarified in the Discussion (lines 422 – 430).

In addition, we ran a multinomial logistic regression using only meanRR (which indirectly reflects heart rate). Its performance was markedly lower than that of models combining several HRV indices, particularly for the C60 and INT conditions, with sensitivities of 46 and 31% respectively, and specificities of 75 and 69%.

# Reviewer #2:

*Methods*

*1. A figure illustrating the exercise schedule would greatly enhance understanding.*

We thank the reviewer for this suggestion. We have added a schematic figure illustrating the experimental protocol. We hope this enhances the clarity of the study. The figure has been added to the Methods section (line 206).

*2. Given that time of day can influence Heart Rate Variability (HRV), could you please specify the exact time at which the exercise sessions were conducted?*

All exercise sessions were conducted at the same time of day for a given participant. This point has been added in the Methods section (line 110).

*3. Data from the final 4 minutes of each exercise session were extracted for subsequent analysis. Given that HRV measurements can fluctuate significantly over time, what was the specific rationale for selecting only the final 4 minutes for analysis?*

The onset of exercise is characterized by marked transient responses (rapid heart rate acceleration and vagal withdrawal). Therefore, the first two minutes were excluded, and analyses focused on the final four minutes to capture the most representative period of exercise-related constraints on HRV (Blasco-Lafarga et al., 2017^[3]^; Park et al., 2014^[4]^).

*> Time-domain analysis included standard deviation of all NN intervals (SDNN) and root mean square of the successive differences of intervals (RMSSD).
1.The time-domain analysis included SDNN, SDANN, SDNNIDX, pNN50, SDSA, r-MSSD, IRRR, MADRR, TINN, HRV index. [book: Martinez et al: Springer international publishing AG 2017, page 30, Table 3.1]. Why only choose the two items above?*

The selection of SDNN and RMSSD was based on the established recommendations (Task Force of the European Society of Cardiology and the North American Society of Pacing and Electrophysiology, 1996^[5]^). These indices are the most commonly used during exercise and are particularly sensitive to exercise intensity (Michael et al., 2017^[6]^). Other indices were excluded to avoid redundancy, as some are strongly correlated with SDNN and RMSSD (e.g. pNN50, SDSD), or for methodological reasons, since indices such as SDANN, SDNN index, or the HRV triangular index require longer recordings and were therefore not suitable for our protocol (Shaffer & Ginsberg, 2017^[7]^). While Martinez et al. (2017) report indices such as IRRR and MADRR, these measures are derived from specific computational approaches (*e.g.* R coding) and are not part of the standard indices commonly used in exercise contexts. Therefore, we focused on SDNN and RMSSD, which are widely established and directly comparable across studies.

*2.It was better to calculate the correlations between these factors.*

We thank the reviewer for the comment. Following the recommendations of Reviewer 1, the model has been revised and now relies on a multinomial logistic regression penalized with Lasso. This approach incorporates an L1 penalty, which reduces the impact of highly correlated variables and, when appropriate, selects a representative variable from a group of redundant predictors (Friedman et al., 2010^[8]^).

*>In the frequency domain, two spectral components were computed: low frequency (LF, 0.04-0.15 Hz), and high frequency (HF, 0.15-0.4 Hz). …The HRV spectrum was generated using Fast Fourier Transform (FFT) based Welch’s periodogram method with a 170-second window and 50% overlap.
1.According to Martinez et al. (Springer International Publishing AG, 2017, p. 37), the frequency components of HRV include VLF, LF, HF, and ULF. Why are additional frequency bands not defined?*

VLF and ULF were not assessed in this study because their reliable estimation requires longer recordings [*i.e.* over 5 minutes for VLF and approximately 24 hours for ULF; (Shaffer & Ginsberg, 2017)]. With recordings of only 4 minutes, only the LF and HF bands can be interpreted reliably, consistent with established literature guidelines.

*2.Why was a 170-second window with 50% overlap used? This window seems rather narrow.*

Power spectral density was estimated from 4-minute (240-second) recordings using Welch’s periodogram, with three main parameters influencing the results: window length, overlap, and window type (e.g. Hanning, Hamming). An overlap of 50% corresponds to the standard value used in the literature. The window length was set to 170 seconds to achieve a compromise between frequency resolution and variance of the estimate: a window that is too short reduces variance but decreases spectral resolution, whereas a window that is too long improves resolution but increases variance (Singh et al., 2004^[9]^). Several window lengths were tested and did not affect our results, confirming the chosen configuration. There is no perfect window, as the optimal choice depends on the study objective and its influence on the results.

*>The fractal correlation properties of the RR interval time series were also calculated using the short-term nonlinear scaling exponent of DFA (DFA-α1)
1.Nonlinear HRV analysis can include several metrics, such as the Poincaré plot, DFA (α1 and α2), entropy-based complexity measures, recurrence quantification analysis, correlation dimension (D2), and Lyapunov exponents. Why did the authors choose to report only this single metric?*

Our aim was not to include all nonlinear HRV parameters, but to select those most relevant based on the literature. DFA-α1 was chosen due to its well-established relationship with exercise intensity and its suitability for short-duration recordings (Gronwald et al., 2020^[10]^; Hottenrott & Hoos, 2017^[11]^). Furthermore, to avoid redundancy, indices such as SD1 and SD2 were not included because they are correlated with other HRV indices (Sassi et al., 2015^[12]^; Shaffer & Ginsberg, 2017). Other indices were not retained because they require longer recordings, such as DFA-α2, which reflects long-term fluctuations (Peng et al., 1995^[13]^).

*2.3. Statistical analysis*

*>A significance level of 0.05 was used for all tests. Statistical analyses were conducted using JASP (version 0.16.0.0) and R (version 4.2.1).
1.Is a one-tailed or two-tailed test being used?*

A two-tailed test was systematically used.

*2.Which R packages were employed in the analysis?*

Repeated-measures ANOVA were performed using JASP software. Following the recommendation of Reviewer 1, the initial standard logistic regression analyses were replaced with a multinomial logistic regression model, implemented in R using *glmnet* and *caret* packages.

*>Standard logistic regressions were employed…
1.If the outcome YYY is typically binomial, which specific variable does it refer to?*

We thank the reviewer for this insightful comment. Following the request of Reviewer 1, we have replaced the previous analyses with multinomial logistic regression model, using exercise modality as the categorical dependent variable with three levels (C40, C60, and INT).

*2.The Hosmer–Lemeshow (HL) test is a statistical method used to assess the goodness of fit of logistic regression models. The authors might consider to do this.*

As the analysis strategy has been revised and now relies on a multinomial logistic regression penalized with Lasso, the Hosmer-Lemeshow test is no longer applicable to the current version of the analysis. We sincerely appreciate your attention to detail, which helped us reflect on and improve the methodological rigor of the manuscript. We hope the updated analysis addresses your concerns, and we remain grateful for your constructive feedback.

*>Collinearity among independent variables was assessed…
1.why not correlation ?*

In the initial analyses based on standard logistic regression, collinearity was assessed using VIF, which provides more informative evaluation of multivariate dependencies than simple pairwise correlation (Kim, 2019^[14]^). However, as previously mentioned, the strategy now relies on a Lasso-penalized multinomial logistic regression, which is relatively insensitive to highly correlated predictors, typically selecting one representative variable from a group of redundant predictors while shrinking the others toward zero.

*>Backward and stepwise procedures were then applied to the remaining predictors to identify the model with the best McFadden r² and smallest Akaike Information Criterion (AIC).
1.how many independent factor?*

We thank the reviewer for this comment. As we have mentioned earlier, the previous analyses have been replaced with multinomial logistic regression model, and the variable selection procedure has been revised accordingly.

*2.why not use R-square ?*

The coefficient of determination R² assesses the proportion of variance in the dependent variable explained by the independent variables. In logistic regression, where the dependent variable is categorical/binomial, the conventional R² cannot be calculated. Alternative measures, known as “pseudo-R²” statistics, are therefore used, including McFadden’s R², which does not directly measure explained variance but rather evaluates model goodness of fit. However, following the change in our analysis strategy, the R² metric is no longer used in the revised version of the manuscript.

*>The variability of the estimates was examined through a bootstrap procedure.
1.Bootstrap techniques are useful when classical methods break down due to small samples, unknown distributions, difficult-to-derive statistics, or model complexity.*

We thank the reviewer for this comment. In the revised version, the previous bootstrap procedure used to examine the variability of estimates has been removed. Model estimation is now based on the penalized multinomial logistic regression approach described in the Methods section.

*>Repeated-measures analysis of variance (ANOVA)
1.which R packages were used for RM-ANOVA ?*

Repeated-measures ANOVA were performed using JASP software. This has been clarified in the Methods section (line 212).

*>Post hoc tests were performed using Holm’s method for pairwise comparisons
1.pairwise comparisons by table or figure might be provided.*

We thank the reviewer for your comment. The results of the post hoc comparisons, performed using Holm’s method, are now presented in the tables with superscript letters, allowing for a clearer reading of the pairwise comparisons.

*Results
>The most accurate predictive model for distinguishing between the C40 and C60 conditions included three predictors: MeanRR, RMSSD and DFA-ɑ1 (Table 3.A). This model achieved a sensitivity of 95.65% and a specificity of 100% (Table 3.B). The McFadden’s r² was 0.91 (p<0.001), and the AIC was 13.58.
1.the outcome Y for logistic regression was usually binominal. What the outcome for these models ?*

Following the suggestion of Reviewer 1, we have replaced the previous analyses with multinomial logistic regression model, using exercise modality as the categorical dependent variable with three levels (C40, C60, and INT).

*2.the correlation between factors ?*

As mentioned in our previous response, we did not calculate correlations between predictors because multicollinearity was assessed using VIF in the initial logistic regression analyses. However, with the update analysis strategy based on multinomial Lasso, correlations are no longer examined, the L1 penalty inherently addresses issues related to highly correlated predictors.

*3.could the author provided the Receiver Operating Characteristic curve and the AUC do show the good results (a sensitivity of 95.65% and a specificity of 100%)*

Please find below the ROC curve for the initial C40 vs C60 binary logistic regression model (image on the left). The corresponding AUC for this model was 0.994. Since we subsequently replaced the previous models with a multinomial regression approach, we also provide the ROC curves for the final model (image on the right), presented separately for each exercise condition. The AUC values for C40, C60 and INT were 0.987, 0.989 and 0.996 respectively.

*4.The McFadden’s r² was super good (0.91), how to get it ?*

McFadden’s R² is calculated from the ratio between the log-likelihood of the fitted model and that of the null model (intercept only) : R²McFadden = 1 – [ln(L_model_)-ln(L_null model_)].

*>Table 4, 5
1.the same question as above.*

The responses provided for table 3 also apply to Tables 4 and 5.

*>Table 6
1.ANOVA could explore the differences between groups, but hard to do regression.*

We agree, the two tests are complementary, but they provide different information, which justifies performing both.

*Discussion
>These predictive properties are supported by statistically significant differences in HRV components across exercise modalities.
1.which factor is significant ?*

We performed one-way ANOVA, so we determined the effect of the exercise modality, without identifying the proper effect of intensity or pattern. It is important to note that a variable that does not show significant differences with ANOVA may still have predictive properties in logistic regression.

# Reviewer #3:

*Abstract
The wording of the Abstract may benefit from clarification and moderation. While the effects of exercise intensity and pattern on HRV metrics are explored through comparisons between exercise modalities (e.g., C40 vs. C60; INT vs. C40–C60), statements implying independent effects of these factors should be aligned more closely with the analytical approach, which does not formally model intensity and pattern as independent factors.*

We thank the reviewer for this comment. We have provided clarifications as suggested, which we hope address the concern.

*Introduction
1) The title is informative but somewhat lengthy. Consider shortening it to improve clarity and readability.*

In light of your comment and that of Reviewer 1, we have decided to modify and shorten it.

*2) Consider streamlining the Introduction by beginning with the discussion at lines 47–49, which directly addresses the use of HRV and wearable devices to detect exercise modalities. This would help sharpen the focus on the central objectives of the study and avoid a potentially distracting opening.*

We thank the reviewer for this relevant comment. Your comment is in line with that of Reviewer 1. The first paragraph of the introduction in the previous version has been moved to the Discussion section (lines 333 – 336).

*3) The description of a “quasi-exponential decrease” in conventional HRV parameters is difficult to interpret and would benefit from additional clarification and supporting references.*

We acknowledge that this term, which has been used in the literature, may be difficult to interpret. The sentence has therefore been revised in the Introduction, and additional references have been added (lines 63 – 65).

*4) Lines 87–89: This comparative statement regarding findings from Cottin et al. is more interpretive in nature and would be better placed in the Discussion rather than the Introduction.*

We thank the reviewer for this comment. The sentence has therefore been removed.

*Methods
1)The manuscript does not indicate whether an a priori or post hoc power analysis was performed to justify the sample size. Please clarify whether the study was adequately powered to detect differences across exercise modalities.*

We acknowledge your remark regarding effect size. No *a priori* effect-size test was conducted in the present study because our primary objective was to generate sufficient heterogeneity to induce distinct physiological adaptations across exercise conditions and to evaluate the predictive capacity of the variables. In this context, the sample size appeared adequate, as the different exercise conditions elicited significant changes in V̇O₂ and blood lactate concentrations, and the ANOVAs revealed significant between-condition differences. In addition, partial effect sizes (η²p) for the ANOVAs ranged from 0.13 to 0.80. Most effects were large (>0.18), except for one at 0.13, which represents a medium effect (0.01 = small; 0.06 = medium; ≥0.14 = large) (Cohen, 2013^[15]^; Lakens, 2013^[16]^). Taken together, these results support the adequacy of the number of subjects included in the study.

*2) In Table 1, reporting both mean ± standard deviation and range (min–max) may be redundant. Unless the range is specifically informative, presenting only one summary measure may improve clarity.*

In our view, these indicators provide different yet complementary information. The mean and standard deviation describe the central tendency and the dispersion around it, whereas the range identify the full span of the observed values, and to verify the absence of unexpected extreme values, which is increasingly encouraged in scientific reporting. Nevertheless, we remain fully open to removing this information should the reviewer consider it preferable, although doing so would inevitably entail a loss of information.

*3) Lines 147-154: For consistency with standard notation, Vmax should be written with “max” as a subscript throughout the manuscript.*

The modifications have been made.

*4) Line 168: Please consider revising the sentence to properly cite the software, for example: “The processed RR interval files were imported into Kubios HRV software (Standard Version 3.5.0; Kubios Oy, Kuopio, Finland) for analysis (Tarvainen et al., XXXX).”*

The citation of Kubios HRV has been updated.

*5) The statistical analysis requires clarification. The manuscript does not specify the type of repeated-measures ANOVA used (e.g., one- vs. two-way), the within-subject factors (exercise modality), or whether interaction effects were tested. In addition, while normality testing and non-parametric alternatives are mentioned, it is unclear what was tested for normality (e.g., which differences). Overall, it may be preferable to adopt a consistent statistical strategy rather than mixing parametric and non-parametric tests across variables. Given that some HRV indices are not normally distributed, a common approach is to apply a logarithmic transformation prior to ANOVA to better meet normality assumptions, or alternatively to apply a fully non-parametric approach consistently across variables.*
As requested, we have clarified the type of ANOVA used, as well as the procedure for verifying normality, in the Methods section (lines 235 – 238).

Following your comment and that of Reviewer 1, we applied a logarithmic transformation to variables that did not satisfy the normality assumption, in order to better approximate normality and thereby justify the exclusive use of parametric ANOVAs. This modification has been added to the Methods section (line 239). Analyses performed after the transformation yielded the same results.

*In continuation of this concern, the Results (Table 6) indicate that a one-way repeated-measures ANOVA was used with exercise modality (C40, C60, INT) as a single within-subject factor. Post hoc pairwise comparisons were then interpreted as reflecting differences in exercise intensity (C40 vs. C60) or exercise pattern (INT vs C40-C60). While appropriate for exploratory condition-based comparisons, this approach does not permit formal testing of exercise pattern and intensity as independent factors, nor their interaction. This limitation should be clearly acknowledged and reflected in the interpretation of the findings.*

We thank the reviewer for this highly relevant comment. We have addressed this point by adding a paragraph in the Discussion (lines 413 – 419) highlighting this limitation and making the interpretation of differences between modalities more cautious. We would also clarify that the study was exploratory, and that the aim was not specifically to test the effect of intensity or pattern, but to examine different exercise conditions.

*Results
1)The tables do not clearly indicate statistical significance (e.g., by using asterisks or similar notation). Please specify statistically significant differences directly in the tables to improve clarity for the reader.*

We thank the reviewer for this suggestion. Superscript letters indicating significant differences between conditions have been added to the tables, and the legends have been updated accordingly.

*Discussion
1) The first paragraph of the Discussion would benefit from being more concise and more closely aligned with the stated objectives in the Introduction. While the Introduction clearly defines the twofold aim of predicting exercise modalities using HRV and identifying modality-specific HRV components, the opening of the Discussion could be streamlined to reflect these objectives more directly. In addition, the opening statement would benefit from more cautious wording, as the analyses demonstrate discrimination between inspected exercise modalities but do not formally test exercise pattern and intensity as independent factors. Clarifying this distinction would improve coherence between the Introduction, analytical approach, and interpretation of the findings.*

We thank the reviewer for this helpful comment. The first paragraph of the Discussion (lines 295 – 299) has been revised to be more concise and more closely aligned with the objectives defined in the Introduction.

*2)The discussion emphasizes very high predictive performance of the logistic regression models (sensitivities and specificities often exceeding 90%). Given the exploratory nature of the study and the relatively small sample size, these results should be interpreted more cautiously.*

In the original version, we emphasized the high predictive performance of our logistic regression models. However, we agree with this comment and have clarified the Discussion to use more cautious wording. For information, following the recommendations of Reviewer 1, the analysis strategy has been revised, and the initial binary logistic regressions were replaced by a multinomial logistic regression penalized with Lasso (that gives similar predictive performance).

*3) The prominent role of MeanRR warrants clarification, as MeanRR reflects average heart rate rather than heart rate variability per se. The discussion should clarify the added value of HRV metrics beyond heart rate alone.*

This comment echoes comment #10 from Reviewer 1. We thank the reviewer for raising this point.

Although meanRR is not a variability metric in itself, its inclusion is methodologically justified because all HRV measures are mathematically derived from the series of RR intervals, and meanRR (inversely proportional to heart rate) reflects the baseline chronotropic state that can condition the interpretation of HRV metrics. Specifically, the average RR interval provides essential contextual information about physiological status even if it does not itself express the structure of variability. Thus, meanRR used as a covariate in a multidimensional domain analysis is fully appropriate. Our results show a good predictive capacity in logit when this variable is associated with temporal and non‑linear indices of HRV. This point has been clarified in the Discussion (lines 422 – 430).

In addition, we ran a multinomial logistic regression using only meanRR (which indirectly reflects heart rate). Its performance was markedly lower than that of models combining several HRV indices, particularly for the C60 and INT conditions, with sensitivities of 46 and 31% respectively, and specificities of 75 and 69%.

*4)External validity: The generalizability of the findings beyond cycling-based exercise and controlled laboratory settings could be briefly discussed.*

In line with the previous comment (comment 2), we agree that external validation is necessary. This point has been added to the Discussion (lines 440 – 443).

*Limitations
Given known sex-related differences in HRV, exploring sex-specific effects may be regarded as a valuable future research direction.*
We thank the reviewer for this comment. A sentence has been added to the Discussion (lines 444 – 445).

*References
The reference list includes several older, foundational studies. Where appropriate, the authors may consider adding more recent literature to complement these references and provide an updated context.*

We acknowledge that the bibliography includes some older articles. Several of them, however, are key references in their fields, such as Astrand & Ryhming (1954) and the HRV Task Force recommendations (1996). The selection of publications is closely aligned with our research question, and the older articles are cited repeatedly because they provide specific elements (for example, references 8–11). Whenever possible, we incorporated more recent studies to update the literature review. In this regard, more than one-third of the cited references were published after 2015.

1. Riddell, M. C., Gallen, I. W., Smart, C. E., Taplin, C. E., Adolfsson, P., Lumb, A. N., Kowalski, A., Rabasa-Lhoret, R., McCrimmon, R. J., Hume, C., Annan, F., Fournier, P. A., Graham, C., Bode, B., Galassetti, P., Jones, T. W., Millán, I. S., Heise, T., Peters, A. L., … Laffel, L. M. (2017). Exercise management in type 1 diabetes: A consensus statement. *The Lancet Diabetes & Endocrinology*, *5*(5), 377–390. <https://doi.org/10.1016/S2213-8587(17)30014->1
2. Reddy, R., Wittenberg, A., Castle, J. R., El Youssef, J., Winters-Stone, K., Gillingham, M., & Jacobs, P. G. (2019). Effect of aerobic and resistance exercise on glycemic control in adults with type 1 diabetes. *Canadian Journal of Diabetes*, *43*(6), 406-414.e1. https://doi.org/10.1016/j.jcjd.2018.08.193
3. Blasco-Lafarga, C., Camarena, B., & Mateo-March, M. (2017). Cardiovascular and Autonomic Responses to a Maximal Exercise Test in Elite Youngsters. *International Journal of Sports Medicine*, *38*(9), 666–674. https://doi.org/10.1055/s-0043-110680
4. Park, S. W., Brenneman, M., Cooke, W. H., Cordova, A., & Fogt, D. (2014). Determination of Anaerobic Threshold by Heart Rate or Heart Rate Variability using Discontinuous Cycle Ergometry. *International Journal of Exercise Science*, *7*(1), 45–53.
5. Task Force of the European Society of Cardiology and the North American Society of Pacing and Electrophysiology. (1996). Heart rate variability. Standards of measurement, physiological interpretation, and clinical use. Task Force of the European Society of Cardiology and the North American Society of Pacing and Electrophysiology. *European Heart Journal*, *17*(3). https://pubmed.ncbi.nlm.nih.gov/8737210/
6. Michael, S., Graham, K. S., & Davis, G. M. (2017). Cardiac Autonomic Responses during Exercise and Post-exercise Recovery Using Heart Rate Variability and Systolic Time Intervals—A Review. *Frontiers in Physiology*, *8*, 301. https://doi.org/10.3389/fphys.2017.00301
7. Shaffer, F., & Ginsberg, J. P. (2017). An Overview of Heart Rate Variability Metrics and Norms. *Frontiers in Public Health*, *5*, 258. https://doi.org/10.3389/fpubh.2017.00258
8. Friedman, J., Hasties, T., & Tibshirani, R. (2010). Regularization Paths for Generalized Linear Models via Coordinate Descent. Journal of Statistical Software, 33(1), 1-22.
9. Singh, D., Vinod, K., Saxena, S. C., & Deepak, K. K. (2004). Effects of RR segment duration on HRV spectrum estimation. *Physiological Measurement*, *25*(3), 721–735. https://doi.org/10.1088/0967-3334/25/3/012
10. Gronwald, T., Rogers, B., & Hoos, O. (2020). Fractal Correlation Properties of Heart Rate Variability: A New Biomarker for Intensity Distribution in Endurance Exercise and Training Prescription? *Frontiers in Physiology*, *11*, 550572. https://doi.org/10.3389/fphys.2020.550572
11. Hottenrott, K., & Hoos, O. (2017). Heart Rate Variability Analysis in Exercise Physiology. In *ECG Time Series Variability Analysis*. CRC Press.
12. Sassi, R., Cerutti, S., Lombardi, F., Malik, M., Huikuri, H. V., Peng, C.-K., Schmidt, G., & Yamamoto, Y. (2015). Advances in heart rate variability signal analysis: Joint position statement by the e-Cardiology ESC Working Group and the European Heart Rhythm Association co-endorsed by the Asia Pacific Heart Rhythm Society. *Europace: European Pacing, Arrhythmias, and Cardiac Electrophysiology: Journal of the Working Groups on Cardiac Pacing, Arrhythmias, and Cardiac Cellular Electrophysiology of the European Society of Cardiology*, *17*(9), 1341–1353. https://doi.org/10.1093/europace/euv015
13. Peng, C. K., Havlin, S., Stanley, H. E., & Goldberger, A. L. (1995). Quantification of scaling exponents and crossover phenomena in nonstationary heartbeat time series. *Chaos (Woodbury, N.Y.)*, *5*(1), 82–87. https://doi.org/10.1063/1.166141
14. Kim, J. H. (2019). Multicollinearity and misleading statistical results. *Korean Journal of Anesthesiology*, *72*(6), 558–569. https://doi.org/10.4097/kja.19087
15. Cohen, J. (2013). *Statistical Power Analysis for the Behavioral Sciences* (2nd ed.). Routledge. https://doi.org/10.4324/9780203771587
16. Lakens, D. (2013). Calculating and reporting effect sizes to facilitate cumulative science: A practical primer for t-tests and ANOVAs. *Frontiers in Psychology*, *4*. https://doi.org/10.3389/fpsyg.2013.00863
