# Supplementary material for: Investigating HRV’s contribution to identify exercise pattern and intensity in healthy subjects
Source: PLoS One. 2026 Jun 11;21(6):e0350856. doi: 10.1371/journal.pone.0350856 (PMC13258023; doi:10.1371/journal.pone.0350856)
Supplement: Peer review history

# Peer review: Decision Letter 0

**DOI:** 10.1371/journal.pone.0350856.r001

**Authors:** Nejka Potocnik

23 Dec 2025

PONE-D-25-48905

From beats to modalities: Investigating HRV’s contribution to exercise differentiation in healthy subjects

PLOS One

Dear Dr. Sanchez,

Thank you for submitting your manuscript to PLOS ONE. After careful consideration, we feel that it has merit but does not fully meet PLOS ONE’s publication criteria as it currently stands. Therefore, we invite you to submit a revised version of the manuscript that addresses the points raised during the review process.

The manuscript investigates a relevant and promising application of HRV for identifying exercise modalities. While the overall approach is interesting, several conceptual and methodological clarifications are needed for the manuscript to meet the PLOS ONE publication criteria. I strongly believe that addressing the issues raised by the reviewers would strengthen the manuscript and its scientific soundness. I encourage the authors to respond to or comment on all questions and suggestions raised by the reviewers.

Please submit your revised manuscript by Feb 06 2026 11:59PM. If you will need more time than this to complete your revisions, please reply to this message or contact the journal office at plosone@plos.org. When you're ready to submit your revision, log on to https://www.editorialmanager.com/pone/ and select the 'Submissions Needing Revision' folder to locate your manuscript file.

Please include the following items when submitting your revised manuscript:

- A letter that responds to each point raised by the academic editor and reviewer(s). You should upload this letter as a separate file labeled 'Response to Reviewers'.
- A marked-up copy of your manuscript that highlights changes made to the original version. You should upload this as a separate file labeled 'Revised Manuscript with Track Changes'.
- An unmarked version of your revised paper without tracked changes. You should upload this as a separate file labeled 'Manuscript'.

If you would like to make changes to your financial disclosure, please include your updated statement in your cover letter. Guidelines for resubmitting your figure files are available below the reviewer comments at the end of this letter.

If applicable, we recommend that you deposit your laboratory protocols in protocols.io to enhance the reproducibility of your results. Protocols.io assigns your protocol its own identifier (DOI) so that it can be cited independently in the future. For instructions see: https://journals.plos.org/plosone/s/submission-guidelines#loc-laboratory-protocols. Additionally, PLOS ONE offers an option for publishing peer-reviewed Lab Protocol articles, which describe protocols hosted on protocols.io. Read more information on sharing protocols at https://plos.org/protocols?utm_medium=editorial-email&utm_source=authorletters&utm_campaign=protocols.

We look forward to receiving your revised manuscript.

Kind regards,

Nejka Potocnik

Academic Editor

PLOS One

Journal requirements:

When submitting your revision, we need you to address these additional requirements.

1. Please ensure that your manuscript meets PLOS ONE's style requirements, including those for file naming. The PLOS ONE style templates can be found at

https://journals.plos.org/plosone/s/file?id=wjVg/PLOSOne_formatting_sample_main_body.pdf and

https://journals.plos.org/plosone/s/file?id=ba62/PLOSOne_formatting_sample_title_authors_affiliations.pdf

2. Thank you for stating the following financial disclosure:

“This study was funded by the University of Montpellier as part of the Companies and Campus program.”

Please state what role the funders took in the study.  If the funders had no role, please state: "The funders had no role in study design, data collection and analysis, decision to publish, or preparation of the manuscript."

If this statement is not correct you must amend it as needed.

Please include this amended Role of Funder statement in your cover letter; we will change the online submission form on your behalf.

3. Thank you for stating the following in the Funding Section of your manuscript:

“This study was funded by the University of Montpellier as part of the Companies and Campus program.”

We note that you have provided funding information that is currently declared in your Funding Statement. However, funding information should not appear in the Acknowledgments section or other areas of your manuscript. We will only publish funding information present in the Funding Statement section of the online submission form.

Please remove any funding-related text from the manuscript and let us know how you would like to update your Funding Statement. Currently, your Funding Statement reads as follows:

“This study was funded by the University of Montpellier as part of the Companies and Campus program.”

Please include your amended statements within your cover letter; we will change the online submission form on your behalf.

If the reviewer comments include a recommendation to cite specific previously published works, please review and evaluate these publications to determine whether they are relevant and should be cited. There is no requirement to cite these works unless the editor has indicated otherwise.

[Note: HTML markup is below. Please do not edit.]

Reviewers' comments:

Reviewer's Responses to Questions

**Comments to the Author**

1. Is the manuscript technically sound, and do the data support the conclusions?

The manuscript must describe a technically sound piece of scientific research with data that supports the conclusions. Experiments must have been conducted rigorously, with appropriate controls, replication, and sample sizes. The conclusions must be drawn appropriately based on the data presented.

Reviewer #1: Partly

Reviewer #2: Partly

Reviewer #3: Partly

**********

2. Has the statistical analysis been performed appropriately and rigorously?

Reviewer #1: No

Reviewer #2: No

Reviewer #3: No

**********

3. Have the authors made all data underlying the findings in their manuscript fully available?

The PLOS Data policy requires authors to make all data underlying the findings described in their manuscript fully available without restriction, with rare exception (please refer to the Data Availability Statement in the manuscript PDF file). The data should be provided as part of the manuscript or its supporting information, or deposited to a public repository. For example, in addition to summary statistics, the data points behind means, medians and variance measures should be available. If there are restrictions on publicly sharing data—e.g. participant privacy or use of data from a third party—those must be specified.

Reviewer #1: Yes

Reviewer #2: No

Reviewer #3: Yes

**********

4. Is the manuscript presented in an intelligible fashion and written in standard English?

PLOS ONE does not copyedit accepted manuscripts, so the language in submitted articles must be clear, correct, and unambiguous. Any typographical or grammatical errors should be corrected at revision, so please note any specific errors here.

Reviewer #1: Yes

Reviewer #2: Yes

Reviewer #3: Yes

**********

5. Review Comments to the Author

Please use the space provided to explain your answers to the questions above. You may also include additional comments for the author, including concerns about dual publication, research ethics, or publication ethics. (Please upload your review as an attachment if it exceeds 20,000 characters)

Reviewer #1: This manuscript aims to determine if HRV components can serve as predictors of "exercise modalities" and to determine which components are influenced by "exercise modality". I believe that the main idea of the paper is helpful, but there are some clarifications and suggestions that I would make the manuscript much stronger.

1) Purpose - Generally speaking, "exercise modalities" refer to the mode under which the exercise is done, such as cycling, running, resistant training, etc. While some may include session pattern or intensity within "modality" I believe that the title could be slightly misleading to the general reader. Maybe specifically referring to "identifying exercise pattern and intensity".

2) Introduction - in the first paragraph, you refer to the influence of exercise on drug pharmacokinetics and then never mention it again. It's not specifically related to the purpose of this paper either. If this is the main motivation, I believe that you should change your purpose statement. If not, this should only be mentioned in the intro or discussion as a practical application of the findings of this manuscript. The introduction reads as though we should be paying close attention to drug pharmacokinetics.

3) The questions from line 47-51 needs clarification. When would clinicians be monitoring exercise and not be able to distinguish between exercise patterns and intensity? Why is HRV a tool that should/could be used here when other tools cannot?

4) When talking about your exercise protocols and MPV protocol, if they have been used elsewhere or are inspired by protocols used elsewhere you need to cite them.

5) 2.2.8 - If you did all of these protocols in the same order, you run into the possibility of your results occurring due to ordered effects. There needs to be an explanation of either why you couldn't counter-order the exercise protocols to prevent this or why it was better to do it the same order every time. Especially with HRV, there is likely to be an ordered effect due to fatigue unless you've controlled for the history with the subjects training every day.

6) 2.3 - A problem with your statistical approach is that you have 2 logistic regression comparisons of exercise pattern, (c40 vs INT & c60 vs INT), but only 1 comparison of intensity (c40 vs c60). This should be consistent. Additionally, I highly recommend conducting a multiple-logistic regression as opposed to three separate logistic regression since each of them are aiming to determine the same purpose and it would make your data stronger.

6.1) 2.3 - You ran the normality after conducting the ANOVA, (it seems), but did you determine the normality of the variables beforehand? Few HRV variables are distributed normally, and therefore most studies apply a natural-log transformation to metrics that violate normality and then run the stats on them. This allows for the inferences to be more robust and strong.

7) On Table 2 and 6 you need to include some form of star or asterisk to denote statistical differences between values.

8) Because of the way that high/short intervals of power can drastically effect the average power numbers, and the physiological response is not well related to this, I would recommend reporting average AND normalized power from each exercise test.

9) Due to the non-normal distribution of HRV metrics, and the few post-hoc p-values from the ANOVA, it seems as though most results did not show significant differences between c40 and INT, though differences were more consistent in c60 vs INT. This seems to call into question the HRV metrics' ability to determine the differences between exercise pattern, in contrast with the logistic regression results. This should be clarified in the discussion.

10) It is very important to address the fact that 2 of your 3 individual logistic regressions had MeanRR interval as the first predictor, and MeanRR is not specifically a measure of heart rate VARIABILITY. The practicality of MeanRR is the same as HR. Additionally, there is already a significant amount of research that shows that HR itself can distinguish between exercise intensity and pattern (when normalized). You should include HR as one of your metrics to determine if HRV can assist in distinguishing between these factors of exercise OVER AND ABOVE HR, and then convince us why HRV is important as a separate predictor/determinant of exercise pattern and intensity.

I believe that these changes will strengthen the findings of this manuscript significantly. Thank you for your submission. I look forward to reading your revisions, as this manuscript was interesting!

Reviewer #2: Topic: From beats to modalities: Investigating HRV’s contribution to exercise 2 differentiation in healthy subjects

I had read this manuscript. The author explore the tool for distinguishing between exercise modalities according to their intensity and continuous or intermittent pattern.

Abstract

HRV was analyzed using time-domain, frequency-domain, and nonlinear measures from data recorded with a Polar H10 Heart Rate Sensor chest strap and processed with Kubios HRV software.

---

Introduction

Thus, the objectives of this study were twofold: i) to explore whether HRV components could serve as predictors of exercise modalities; and ii) to pinpoint components in the time and frequency domains, as well as nonlinear measures that are specifically influenced by exercise modalities, focusing on the exercise pattern (continuous vs. intermittent) and its intensity.

2. Methods

Table 1 presented the basic data.

> Three distinct exercise modalities were evaluated, each lasting 6 minutes.

1. A figure illustrating the exercise schedule would greatly enhance understanding.

2. Given that time of day can influence Heart Rate Variability (HRV), could you please specify the exact time at which the exercise sessions were conducted?

3. Data from the final 4 minutes of each exercise session were extracted for subsequent analysis. Given that HRV measurements can fluctuate significantly over time, what was the specific rationale for selecting only the final 4 minutes for analysis?

> Time-domain analysis included standard deviation of all NN intervals (SDNN) and root mean square of the successive differences of intervals (RMSSD).

1.The time-domain analysis included SDNN, SDANN, SDNNIDX, pNN50, SDSA, r-MSSD, IRRR, MADRR, TINN, HRV index. [book: Martinez et al: Springer international publishing AG 2017, page 30, Table 3.1]

Why only choose the two items above?

2.It was better to calculate the correlations between these factors.

>In the frequency domain, two spectral components were computed: low frequency (LF, 0.04-0.15 Hz), and high frequency (HF, 0.15-0.4 Hz). …The HRV spectrum was generated using Fast Fourier Transform (FFT) based Welch’s

periodogram method with a 170-second window and 50% overlap.

1.According to Martinez et al. (Springer International Publishing AG, 2017, p. 37), the frequency components of HRV include VLF, LF, HF, and ULF. Why are additional frequency bands not defined?

2.Why was a 170-second window with 50% overlap used? This window seems rather narrow.

>The fractal correlation properties of the RR interval time series were also calculated using the short-term nonlinear scaling exponent of DFA (DFA-α1)

1.Nonlinear HRV analysis can include several metrics, such as the Poincaré plot, DFA (α1 and α2), entropy-based complexity measures, recurrence quantification analysis, correlation dimension (D2), and Lyapunov exponents. Why did the authors choose to report only this single metric?

2.3. Statistical analysis

>A significance level of 0.05 was used for all tests. Statistical analyses were conducted using JASP (version 0.16.0.0) and R (version 4.2.1).

1.Is a one-tailed or two-tailed test being used?

2.Which R packages were employed in the analysis?

>Standard logistic regressions were employed…

1.If the outcome YYY is typically binomial, which specific variable does it refer to?

2.The Hosmer–Lemeshow (HL) test is a statistical method used to assess the goodness of fit of logistic regression models. The authors might consider to do this.

>Collinearity among independent variables was assessed…

1.why not correlation ?

>Backward and stepwise procedures were then applied to the remaining predictors to identify the model with the best McFadden r² and smallest Akaike Information Criterion (AIC).

1.how many independent factor?

2.why not use R-square ?

>The variability of the estimates was examined through a bootstrap procedure.

1.Bootstrap techniques are useful when classical methods break down due to small samples, unknown distributions, difficult-to-derive statistics, or model complexity.

>Repeated-measures analysis of variance (ANOVA)

1.which R packages were used for RM-ANOVA ?

>Post hoc tests were performed using Holm’s method for pairwise comparisons

1.pairwise comparisons by table or figure might be provided.

Results

>The most accurate predictive model for distinguishing between the C40 and C60 conditions included three predictors: MeanRR, RMSSD and DFA-ɑ1 (Table 3.A). This model achieved a sensitivity of 95.65% and a specificity of 100% (Table 3.B). The McFadden’s r² was 0.91 (p<0.001), and the AIC was 13.58.

1.the outcome Y for logistic regression was usually binominal. What the outcome for these models ?

2.the correlation between factors ?

3.could the author provided the Receiver Operating Characteristic curve and the AUC do show the good results (a sensitivity of 95.65% and a specificity of 100%) ?

4.The McFadden’s r² was super good (0.91), how to get it ?

>Table 4, 5

1.the same question as above.

>Table 6

1.ANOVA could explore the differences between groups, but hard to do regression.

Discussion

>These predictive properties are supported by statistically significant differences in HRV components across exercise modalities.

1.which factor is significant ?

Reviewer #3: The manuscript investigates a relevant and promising application of HRV for identifying exercise modalities. While the manuscript is clearly written and the overall approach is interesting, several conceptual and methodological clarifications would strengthen the manuscript and its scientific soundness. Here are my comments:

Abstract

The wording of the Abstract may benefit from clarification and moderation. While the effects of exercise intensity and pattern on HRV metrics are explored through comparisons between exercise modalities (e.g., C40 vs. C60; INT vs. C40–C60), statements implying independent effects of these factors should be aligned more closely with the analytical approach, which does not formally model intensity and pattern as independent factors.

Introduction

1) The title is informative but somewhat lengthy. Consider shortening it to improve clarity and readability.

2) Consider streamlining the Introduction by beginning with the discussion at lines 47–49, which directly addresses the use of HRV and wearable devices to detect exercise modalities. This would help sharpen the focus on the central objectives of the study and avoid a potentially distracting opening.

3) The description of a “quasi-exponential decrease” in conventional HRV parameters is difficult to interpret and would benefit from additional clarification and supporting references.

4) Lines 87–89: This comparative statement regarding findings from Cottin et al. is more interpretive in nature and would be better placed in the Discussion rather than the Introduction.

Methods

1)The manuscript does not indicate whether an a priori or post hoc power analysis was performed to justify the sample size. Please clarify whether the study was adequately powered to detect differences across exercise modalities.

2) In Table 1, reporting both mean ± standard deviation and range (min–max) may be redundant. Unless the range is specifically informative, presenting only one summary measure may improve clarity.

3) Lines 147-154: For consistency with standard notation, Vmax should be written with “max” as a subscript throughout the manuscript.

4) Line 168: Please consider revising the sentence to properly cite the software, for example: “The processed RR interval files were imported into Kubios HRV software (Standard Version 3.5.0; Kubios Oy, Kuopio, Finland) for analysis (Tarvainen et al., XXXX).”

5) The statistical analysis requires clarification. The manuscript does not specify the type of repeated-measures ANOVA used (e.g., one- vs. two-way), the within-subject factors (exercise modality), or whether interaction effects were tested. In addition, while normality testing and non-parametric alternatives are mentioned, it is unclear what was tested for normality (e.g., which differences). Overall, it may be preferable to adopt a consistent statistical strategy rather than mixing parametric and non-parametric tests across variables. Given that some HRV indices are not normally distributed, a common approach is to apply a logarithmic transformation prior to ANOVA to better meet normality assumptions, or alternatively to apply a fully non-parametric approach consistently across variables.

In continuation of this concern, the Results (Table 6) indicate that a one-way repeated-measures ANOVA was used with exercise modality (C40, C60, INT) as a single within-subject factor. Post hoc pairwise comparisons were then interpreted as reflecting differences in exercise intensity (C40 vs. C60) or exercise pattern (INT vs C40-C60). While appropriate for exploratory condition-based comparisons, this approach does not permit formal testing of exercise pattern and intensity as independent factors, nor their interaction. This limitation should be clearly acknowledged and reflected in the interpretation of the findings.

Results

1)The tables do not clearly indicate statistical significance (e.g., by using asterisks or similar notation). Please specify statistically significant differences directly in the tables to improve clarity for the reader.

Discussion

1) The first paragraph of the Discussion would benefit from being more concise and more closely aligned with the stated objectives in the Introduction. While the Introduction clearly defines the twofold aim of predicting exercise modalities using HRV and identifying modality-specific HRV components, the opening of the Discussion could be streamlined to reflect these objectives more directly. In addition, the opening statement would benefit from more cautious wording, as the analyses demonstrate discrimination between inspected exercise modalities but do not formally test exercise pattern and intensity as independent factors. Clarifying this distinction would improve coherence between the Introduction, analytical approach, and interpretation of the findings.

2)The discussion emphasizes very high predictive performance of the logistic regression models (sensitivities and specificities often exceeding 90%). Given the exploratory nature of the study and the relatively small sample size, these results should be interpreted more cautiously.

3) The prominent role of MeanRR warrants clarification, as MeanRR reflects average heart rate rather than heart rate variability per se. The discussion should clarify the added value of HRV metrics beyond heart rate alone.

4)External validity: The generalizability of the findings beyond cycling-based exercise and controlled laboratory settings could be briefly discussed.

Limitations

Given known sex-related differences in HRV, exploring sex-specific effects may be regarded as a valuable future research direction.

References

The reference list includes several older, foundational studies. Where appropriate, the authors may consider adding more recent literature to complement these references and provide an updated context.

**********

6. PLOS authors have the option to publish the peer review history of their article (what does this mean?). If published, this will include your full peer review and any attached files.

If you choose “no”, your identity will remain anonymous but your review may still be made public.

**Do you want your identity to be public for this peer review?**   For information about this choice, including consent withdrawal, please see our Privacy Policy.

Reviewer #1: **Yes:**  Andrew D. Fields

Reviewer #2: No

Reviewer #3: No

**********

[NOTE: If reviewer comments were submitted as an attachment file, they will be attached to this email and accessible via the submission site. Please log into your account, locate the manuscript record, and check for the action link "View Attachments". If this link does not appear, there are no attachment files.]

To ensure your figures meet our technical requirements, please review our figure guidelines: https://journals.plos.org/plosone/s/figures

You may also use PLOS’s free figure tool, NAAS, to help you prepare publication quality figures: https://journals.plos.org/plosone/s/figures#loc-tools-for-figure-preparation.

NAAS will assess whether your figures meet our technical requirements by comparing each figure against our figure specifications.

# Author comment: Author response to Decision Letter 1

**DOI:** 10.1371/journal.pone.0350856.r002

15 Apr 2026

Please see the attached "Response to Reviewers" document for our detailed point-by-point responses to the reviewers' comments. We have also uploaded an additional cover letter including the requested clarification regarding funding.

Attachment Submitted filename: Response to Reviewers.docx

# Peer review: Decision Letter 1

**DOI:** 10.1371/journal.pone.0350856.r003

**Authors:** Nejka Potocnik

19 May 2026

Investigating HRV’s contribution to identify exercise pattern and intensity in healthy subjects

PONE-D-25-48905R1

Dear Dr. Sanchez,

We’re pleased to inform you that your manuscript has been judged scientifically suitable for publication and will be formally accepted for publication once it meets all outstanding technical requirements.

Within one week, you’ll receive an e-mail detailing the required amendments. When these have been addressed, you’ll receive a formal acceptance letter and your manuscript will be scheduled for publication.

An invoice will be generated when your article is formally accepted. Please note, if your institution has a publishing partnership with PLOS and your article meets the relevant criteria, all or part of your publication costs will be covered. Please make sure your user information is up-to-date by logging into Editorial Manager at Editorial Manager® and clicking the ‘Update My Information' link at the top of the page. For questions related to billing, please contact billing support.

If your institution or institutions have a press office, please notify them about your upcoming paper to help maximize its impact. If they’ll be preparing press materials, please inform our press team as soon as possible -- no later than 48 hours after receiving the formal acceptance. Your manuscript will remain under strict press embargo until 2 pm Eastern Time on the date of publication. For more information, please contact onepress@plos.org.

Kind regards,

Nejka Potocnik

Academic Editor

PLOS One

Additional Editor Comments (optional):

Reviewers' comments:

Reviewer's Responses to Questions

**Comments to the Author**

1. If the authors have adequately addressed your comments raised in a previous round of review and you feel that this manuscript is now acceptable for publication, you may indicate that here to bypass the “Comments to the Author” section, enter your conflict of interest statement in the “Confidential to Editor” section, and submit your "Accept" recommendation.

Reviewer #1: All comments have been addressed

Reviewer #3: All comments have been addressed

**********

2. Is the manuscript technically sound, and do the data support the conclusions?

The manuscript must describe a technically sound piece of scientific research with data that supports the conclusions. Experiments must have been conducted rigorously, with appropriate controls, replication, and sample sizes. The conclusions must be drawn appropriately based on the data presented.

Reviewer #1: Yes

Reviewer #3: Yes

**********

3. Has the statistical analysis been performed appropriately and rigorously?

Reviewer #1: Yes

Reviewer #3: Yes

**********

4. Have the authors made all data underlying the findings in their manuscript fully available?

The PLOS Data policy requires authors to make all data underlying the findings described in their manuscript fully available without restriction, with rare exception (please refer to the Data Availability Statement in the manuscript PDF file). The data should be provided as part of the manuscript or its supporting information, or deposited to a public repository. For example, in addition to summary statistics, the data points behind means, medians and variance measures should be available. If there are restrictions on publicly sharing data—e.g. participant privacy or use of data from a third party—those must be specified.

Reviewer #1: Yes

Reviewer #3: Yes

**********

5. Is the manuscript presented in an intelligible fashion and written in standard English?

PLOS ONE does not copyedit accepted manuscripts, so the language in submitted articles must be clear, correct, and unambiguous. Any typographical or grammatical errors should be corrected at revision, so please note any specific errors here.

Reviewer #1: Yes

Reviewer #3: Yes

**********

6. Review Comments to the Author

Please use the space provided to explain your answers to the questions above. You may also include additional comments for the author, including concerns about dual publication, research ethics, or publication ethics. (Please upload your review as an attachment if it exceeds 20,000 characters)

Reviewer #1: All of my comments and suggestions have been sufficiently addressed. This is a great manuscript! You should be proud.

Reviewer #3: I would like to thank the authors for their thorough responses to my comments. I appreciate the substantial revisions made throughout the manuscript, which have significantly enhanced its overall clarity and rigor.

In particular, I find that the manuscript has been notably strengthened through the improvements to the statistical analysis and the additional methodological and interpretative clarifications provided in the revised version. The authors have addressed the concerns regarding the analytical approach in a careful and transparent manner, and the discussion now reflects the exploratory nature of the study more appropriately.

I also find the revised title clearer and more concise than the original version. In addition, the Discussion section is now better introduced, and the added introductory paragraph improves the coherence between the study objectives, analyses, and interpretation of the findings. I also appreciate mentioning the external validity and potential real-life applicability of the findings, particularly in the context of wearable-device monitoring and clinical population

Overall, I believe the manuscript has improved considerably following revision and is now suitable for publication in its current form.

With best regards,

Reviewer

**********

7. PLOS authors have the option to publish the peer review history of their article (what does this mean?). If published, this will include your full peer review and any attached files.

If you choose “no”, your identity will remain anonymous but your review may still be made public.

**Do you want your identity to be public for this peer review?**   For information about this choice, including consent withdrawal, please see our Privacy Policy.

Reviewer #1: **Yes:**  Andrew D. Fields

Reviewer #3: **Yes:**  Lana Kralj

**********

# Editor report: Acceptance letter

**DOI:** 10.1371/journal.pone.0350856.r004

**Authors:** Nejka Potocnik

PONE-D-25-48905R1

PLOS One

Dear Dr. Sanchez,

I'm pleased to inform you that your manuscript has been deemed suitable for publication in PLOS One. Congratulations! Your manuscript is now being handed over to our production team.

At this stage, our production department will prepare your paper for publication. This includes ensuring the following:

* All references, tables, and figures are properly cited

* All relevant supporting information is included in the manuscript submission,

* There are no issues that prevent the paper from being properly typeset

You will receive further instructions from the production team, including instructions on how to review your proof when it is ready. Please keep in mind that we are working through a large volume of accepted articles, so please give us a few days to review your paper and let you know the next and final steps.

Lastly, if your institution or institutions have a press office, please let them know about your upcoming paper now to help maximize its impact. If they'll be preparing press materials, please inform our press team within the next 48 hours. Your manuscript will remain under strict press embargo until 2 pm Eastern Time on the date of publication. For more information, please contact onepress@plos.org.

You will receive an invoice from PLOS for your publication fee after your manuscript has reached the completed accept phase. If you receive an email requesting payment before acceptance or for any other service, this may be a phishing scheme. Learn how to identify phishing emails and protect your accounts at https://explore.plos.org/phishing.

If we can help with anything else, please email us at customercare@plos.org.

Thank you for submitting your work to PLOS ONE and supporting open access.

Kind regards,

PLOS ONE Editorial Office Staff

on behalf of

Dr. Nejka Potocnik

Academic Editor

PLOS One
